# Case report: Chlorpromazine and deep venous thrombosis

**DOI:** 10.1108/MIJ-10-2019-0005

**Published:** 2019-11-04

**Authors:** Matthew Joseph Reed, Sean Comeau, Todd R. Wojtanowicz, Bharat Reddy Sampathi, Sofia Penev, Robert Bota

**Affiliations:** Department of Psychiatry, University of California, Irvine, California, USA

**Keywords:** Clozapine, Risk factors, Antipsychotic drugs, Bipolar disorder, Chlorpromazine, Venous thromboembolisms

## Abstract

**Purpose:**

Since the development of antipsychotic drugs in the 1950s, a variety of studies and case reports have been published that suggest an association between exposure to typical antipsychotics and venous thromboembolisms (VTE). Therefore, when starting treatment with antipsychotics, especially low-potency typical antipsychotics and clozapine, health-care providers must account for the patient’s existing VTE risk factors.

**Design/methodology/approach:**

In this case report, the authors describe the development of a pulmonary embolism associated with use of chlorpromazine in the treatment of an acute manic episode in a 51-year-old female patient with bipolar disorder type 1.

**Findings:**

The patient was brought to the emergency room by the police on a legal hold for bizarre behaviors at a bus stop, which included incessantly yelling at bystanders. The patient was found to have disorganized thoughts, poor sleep, rapid speech, labile mood, distractibility, auditory hallucinations and grandiose delusions. During the course of her stay, the patient received extensive IM chlorpromazine for extreme agitation, in addition to chlorpromazine 200 mg IM Q8H, which was later decreased to chlorpromazine 100 mg chlorpromazine IM/PO Q8H. On day 4 of the treatment, the patient experienced difficulty breathing, hypoxia and tachycardia and was found to have bilateral expiratory wheezes. CT angiography showed sub-segmental pulmonary embolus and the patient was transferred to MICU service. The patient was then intubated and started on heparin by the medical team. Over the course of the next day, her respiratory distress resolved and the patient was extubated.

**Originality/value:**

It is possible that chlorpromazine may indeed increase VTEs, and there are various physiological postulations regarding the mechanism of action. However, multiple confounding variables existed in the authors’ report, including venous stasis and the use of restraints, tobacco and valproic acid. Each of these variables has been shown to increase VTE occurrence. Further controlled studies are necessary to identify the true relationship between antipsychotics and VTEs.

## Introduction

Since the development of antipsychotic drugs in the 1950s, a variety of studies and case reports have been published that suggest an association between exposure to typical antipsychotics and venous thromboembolism (VTE). Such an association has also been identified with Clozapine (Walker *et al.*, 1997). VTE includes deep vein thrombosis (DVT) and pulmonary embolism (PE), which can be fatal. Therefore, when starting treatment with antipsychotics, especially low-potency typical antipsychotics and clozapine, health care providers must account for the patient’s existing VTE risk factors. Clinicians should consider interrupting or changing the antipsychotic regimen in patients in whom this adverse effect is suspected, such as in patients with chest pain, dyspnea or oxygen desaturation. More studies are currently required to determine the incidence rate, the possible predisposing factors and the biological mechanisms involved.

In several studies, such as by Parkin *et al.* (2003) and Zornberg and Jick (2000), low-potency antipsychotics, such as the phenothiazines, were shown to be more strongly associated with increased risk for VTE than high-potency antipsychotics, such as haloperidol. Several hypotheses have been proposed for the biological mechanisms by which antipsychotics may portend increased VTE risk, including: elevation of anticardiolipin autoantibodies (Canoso *et al.*, 1990), enhanced platelet aggregation (Zornberg and Jick, 2000) venous stasis caused by sedation and hyperhomocysteinemia (Ray *et al.*, 2002). We cannot exclude the possibility that the underlying psychiatric disorders themselves are associated with thromboembolic phenomena. In fact, this link has been made by a number of studies, which discuss VTE risk factors present in psychiatric disorders, which are independent of pharmacotherapy, such as increased adrenaline secretion during acute psychotic episodes (Hindersin *et al.*, 1984), use of physical restraints (Lazarus, 2001), increased frequency of smoking in patients with schizophrenia (Hughes *et al.*, 1986) as well as abnormal phospholipid metabolism and autoimmunity in schizophrenia (Halacheva *et al.*, 2009).

In this case report, we describe the development of a PE associated with use of chlorpromazine in the treatment of an acute manic episode in a patient with a diagnosis of bipolar disorder type 1.

## Case report

A 51-year-old female with self-reported bipolar disorder presented to the emergency room brought in by police for bizarre behaviors at a bus stop, which included incessantly yelling at bystanders. She was found to have disorganized thoughts, poor sleep, rapid speech, labile mood, distractibility, auditory hallucinations and grandiose delusions. A 5,150 hold was placed and patient was started on olanzapine 10 mg BID, clonazepam 0.5 mg BID and olanzapine 5 mg Q6H PRN for agitation. Of note, the patient had flu-like symptoms and a positive influenza swab and was subsequently started on oseltamivir. The patient was confined to the ED as there were no psychiatric inpatient units that could accommodate droplet precautions at the time. She was also too disorganized to trust her with an infection prevention mask.

Day 2: While in the emergency room, the patient required multiple rounds of emergency medications because of agitation, aggression and inability to redirect from staff on the first night. These included two rounds of haloperidol 5 mg + lorazepam 2 mg + diphenhydramine 50 mg IM, olanzapine 5 mg PO × 1 and chlorpromazine 50 mg IM × 1. Because of no apparent change in her behavior, the standing olanzapine dose was increased to 15 mg PO BID and clonazepam 1 mg PO BID.

Day 3: The patient continued to require multiple rounds of emergency medications as she was not responding to olanzapine 5 mg PRN for agitation with two additional rounds of haloperidol 5 mg + lorazepam 2 mg + diphenhydramine 50 mg IM need overnight. Because of lack of response from IM emergency medication cocktail, the emergency medication recommendation was changed to chlorpromazine 100 mg PO/IM + lorazepam 1 mg PO/IM. Also, valproic acid 500 mg QAM + 1000 mg QHS PO was added for additional mood stabilization.

Day 4: Patient was placed on a 5,250 hold as she continued to appear gravely disabled. Overnight, the patient was reported to be extremely agitated, demanding and not verbally redirectable. Patient required prns of Zyprexa 5 mg × 2 and emergency medications chlorpromazine 100 mg and lorazepam 1 mg IM with no significant effect on her behavior or symptomatology. The emergency medication was changed to offer PO chlorpromazine 200 mg Q8H and lorazepam 1 mg Q8H, if refused then chlorpromazine 200 mg IM Q8H (divided) + lorazepam 1 mg IM Q8H for agitation (2nd line). After 2 days on this regimen, the patient became increasingly somnolent so the regimen was changed back to chlorpromazine 100 mg PO/IM + lorazepam 1 mg PO/IM. However, the patient continued to be increasingly somnolent. She also demonstrated slurred speech, which was thought to be related to emergency medications and poor sleep.

On the day the patient was medically cleared for psychiatric hospitalization, she was reported by the ED nurse to have difficulty breathing in the ED becoming hypoxic and tachycardia. She was found to have bilateral expiratory wheezes on physical examination and was treated with ipratropium bromide 0.5 mg/albuterol sulfate 3.0 mg. Chest X-ray obtained showed possible left-sided pneumonia. Patient was given IV rocephin and azithromycin and fluids. CT angio done to rule out PE and the patient was admitted to medicine for pneumonia.

On admission to medicine, the patient had a valproic acid level of 166 mcg/ml. The patient was noted becoming increasingly agitated with respiratory distress. As she was altered and unable to tolerate BiPAP, she was intubated for airway protection and hypoxic respiratory failure. By this time, the CT angiography showed sub-segmental pulmonary embolus and the patient was transferred to MICU service. There she was determined to have combined hypercarbic and hypoxic respiratory failure secondary to COPD exacerbation with influenza, possible hospital-acquired pneumonia given prolonged hospital stay and possible PE given immobility in ED, tachycardia and high Well’s score. She was then started on heparin by the medical team and over the course of the next day, her respiratory distress resolved and she was extubated.

## Discussion

It has been noted in multiple studies that the specific use of chlorpromazine may increase VTE occurrences. From 1953 to 1977, after the discovery of chlorpromazine’s antipsychotic effects, the literature in Germany indicated that antipsychotic use may lead to fatal PE. In one study from 1954 to 1957, 11 out of 338 phenothiazine users died from PEs compared to just one in the non-phenothiazine group (Masopust *et al.*, 2012). In a more recent study, Zornberg and Jick analyzed 29,952 antipsychotic medication users and calculated an odds ratio of 3.3 for high-potency antipsychotics and 24.1 for low-potency antipsychotics such as chlorpromazine in regards to PE outcomes (Zornberg and Jick, 2000).

Anticardiolipin antibodies have been identified as a possible contributor to thrombosis. Roche-Bayard *et al.* presented a case of chlorpromazine-induced lupus erythematosus that resulted in multiple thromboembolic events and eventually a PE. Laboratory tests for this patient showed increased IgM and IgG anticardiolipin antibodies, antiprothombinase-type circulating anticoagulant, and decreased factors VII, IX and XI (Roche-Bayard *et al.*, 1990). Furthermore, a few studies have indicated that chlorpromazine use can lead to 5-HT induced platelet aggregation. However, these results have since been unreplaceable and later studies reported that noradrenaline increases 5-HT induced aggregation. Therefore, it is possible that the 5-HT enhancement is because of elevated plasma noradrenaline in stressed schizophrenics that were concurrently treated with chlorpromazine (Orr and Boullin, 1976) Venous stasis can also occur in patients on antipsychotics because of the sedative effects of the drugs. Finally, one study has suggested that increased homocysteine levels as a byproduct of antipsychotic use can lead to VTEs as well (Ray *et al.*, 2002).

It is important to identify the variables that confound the relationship between chlorpromazine and VTEs. Reviews have identified a positive correlation between prolonged immobilization from physical restraints and VTE, even without other DVT risk factors (De Hert *et al.*, 2010). Furthermore, chronic smokers are at an increased risk of VTEs, and a study by Hughes *et al.* found that smoking was significantly high in psychiatric patients when compared to a random population-based sample of 1,140 Minnesotans and 17,000 US citizens (52 per cent vs 30 per cent vs 33 per cent, respectively). Among the psychiatric patients, smoking was especially high in mania (70 per cent) (Hughes *et al.*, 1986).

Another possible relationship between chlorpromazine and VTE may include concurrent Depakote use. A study in 2005 concluded that Depakote may potentiate typical and atypical antipsychotics leading to increased prefrontal dopamine release via 5-HT (1 A) receptor activation (Ichikawa *et al.*, 2005). Given past studies stating that chlorpromazine may cause platelet aggregation via 5-HT receptor activation, the use of valproic acid may indirectly increase VTEs in patients with simultaneous antipsychotic use.

Our case report provides important insight into the possible association between chlorpromazine and VTEs and the confounding variables. During the course of her stay, our patient received extensive IM chlorpromazine for extreme agitation in addition to chlorpromazine 200 mg IM Q8H, which was later decreased to chlorpromazine 100 mg chlorpromazine IM/PO Q8H. Given the large cumulative dosing of chlorpromazine during the patient’s hospitalization, it is a possibility that chlorpromazine itself led to the patients VTE based the studies mentioned above. However, the patient was very sedated and in restraints for a lengthy period leading to both prolonged immobility and venous stasis. This may have led to her PE. Furthermore, the patient is a chronic tobacco user, another factor known to increase clotting. Presenting with symptoms of mania, elevated noradrenaline levels could have also contributed to her PE. Finally, if it is true that valproic acid potentiates the effects of antipsychotics, an increased level of 166 mcg/ml may have led to higher chlorpromazine concentrations than desired, contributing to her PE. Retrospectively, it would have been useful to record the patient’s homocysteine level given data that increased homocysteine may cause clotting. It is also important to note that the patient was an extremely poor historian and it is unclear if she had underlying comorbidities contributing to her PE, including hypercholesterolemia and chronic hypertension.

## Conclusion

In this case report, we describe the development of a PE associated with chlorpromazine use in the treatment of an acute manic episode in a bipolar type 1 patient. In conclusion, chlorpromazine may indeed increase VTEs, but multiple variables confound the ability to elucidate the degree of effect. Further controlled studies are needed to make progress in this long-standing debate.
